# T Cell Dysfunction and Exhaustion in Cancer

**DOI:** 10.3389/fcell.2020.00017

**Published:** 2020-02-11

**Authors:** Zhen Zhang, Shasha Liu, Bin Zhang, Liang Qiao, Yi Zhang, Yi Zhang

**Affiliations:** ^1^Biotherapy Center, The First Affiliated Hospital of Zhengzhou University, Zhengzhou, China; ^2^Department of Hematology/Oncology, School of Medicine, Northwestern University, Chicago, IL, United States; ^3^Department of Microbiology and Immunology, Stritch School of Medicine, Health Sciences Division, Loyola University Chicago, Maywood, IL, United States; ^4^Fels Institute for Cancer Research and Molecular Biology, Lewis Katz School of Medicine, Temple University, Philadelphia, PA, United States; ^5^Cancer Center, The First Affiliated Hospital of Zhengzhou University, Zhengzhou, China; ^6^Henan Key Laboratory for Tumor Immunology and Biotherapy, Zhengzhou, China; ^7^School of Life Sciences, Zhengzhou University, Zhengzhou, China

**Keywords:** T cells dysfunction, intrinsic factors, extrinsic factors, tumor microenvironment, cancer immunotherapy

## Abstract

Tumor immunotherapy is a promising therapeutic strategy for patients with advanced cancers. T cells are key mediators of antitumor function that specifically recognize and react to tumor-expressing antigens and have proven critical for cancer immunotherapy. However, T cells are not as effective against cancer as expected. This is partly because T cells enter a dysfunctional or exhausted state, which is characterized by sustained expression of inhibitory receptors and a transcriptional state distinct from that of functional effector or memory T cells. T cell dysfunction induces the out of control of tumors. Recently, T cell dysfunction has been investigated in many experimental and clinical settings. The molecular definition of T cell dysfunction and the underlying causes of the T cell dysfunction has been advanced regardless of the fact that the pathways involved are not well elucidated, which proposing promising therapeutic opportunities in clinic. In this review, we will discuss the recent advances in the molecular mechanisms that affect TME and induce T cell dysfunction, and the development of promising immunotherapies to counteract the mechanisms of tumor-induced T cell dysfunction. Better understanding these underlying mechanisms may lead to new strategies to improve the clinical outcome of patients with cancer.

## Introduction

Cancer immunotherapy is a transformative strategy that utilizes the immune system of the body to treat cancer. T cells destruct tumor cells by recognizing and reacting to tumor-associated antigens through their T cell receptors (TCRs) (Kishton et al., 2017). Considerable progress has been made in the development of immunotherapy techniques that enhance T cell anti-tumor immunity, including adoptive transfer of tumor infiltrating lymphocytes (TILs), endogenous peripheral blood-derived T cells (ETC), chimeric antigen receptor-engineered T cells (CAR-T), and TCR-engineered T cells (TCR-T). In addition, neoantigen vaccines and checkpoint blockade therapies using anti-programed cell death 1 (PD-1) and anti-PD-ligand 1 (PD-L1) have shown potent therapeutic effects in patients with advanced cancer (Chapuis et al., 2016b; Yee and Lizee, 2017; Boyiadzis et al., 2018; Yee, 2018; Riley et al., 2019). These encouraging results demonstrate that T cell-based cancer therapies offer great promise in inducing complete responses in patients against several types of cancer (Maude et al., 2014; Yee et al., 2015). However, many patients who have responded to T cell-based therapies do not achieve durable clinical responses. Mechanisms underlying resistance or short-term response to these therapies remain largely unknown. Some studies suggest that the efficacy of immunotherapy is limited by the generation of dysfunctional T cells in the tumor microenvironment (TME) (Thommen and Schumacher, 2018; Vodnala et al., 2019). Indeed, negative regulators that mediate T cell dysfunction have been identified in mice and humans. For example, treatment with anti-PD-1/PD-L1 and anti-CTLA-4 immune checkpoint inhibitors reinvigorate dysfunctional TILs and augment their anti-tumor effects (Wherry and Kurachi, 2015; Zarour, 2016; Miller et al., 2019; Wang et al., 2019a). Thus, a better understanding of the mechanisms underlying T cell dysfunction in the TME may lead to novel therapeutic interventions for patients with cancer.

Recent studies have shown that the exhaustion and functional impairment of T cells in the TME is a defining feature of many cancers (Jiang et al., 2015). The TME consists of cancer cells as well as immunosuppressive cells and their associated cytokines, i.e., interleukin-10 (IL-10) and transforming growth factor-β (TGF-β) that facilitate tumor progression and mediate T cell dysfunction. Many studies have shown that dysfunctional CD8^+^ T cells in cancer are characterized by high expression levels of inhibitory receptors, including PD-1, TIM-3, LAG-3 and immunoreceptor tyrosine-based inhibitory motif domain (TIGIT), which are positively associated with T cell exhaustion. In addition, these dysfunctional CD8^+^ T cells show impaired production of effector cytokines, such as IL-2, IFN-γ and tumor necrosis factor-α (TNF-α) (Chauvin et al., 2015; Wang et al., 2017). Moreover, T cell dysfunction results in impaired proliferation and decreased production of effector molecules in response to tumor antigens. In this review, we discuss the characteristics of T cell dysfunction in cancer with an aim to elucidate the molecular mechanisms through which TME-derived factors mediate T cell dysfunction factors, and this may promote the exploration of novel strategies for restoring intratumoral T cell function, which can further enhance immunotherapy efficacy.

## Hallmarks of T Cell Dysfunction in Cancer

**FIGURE 1:**
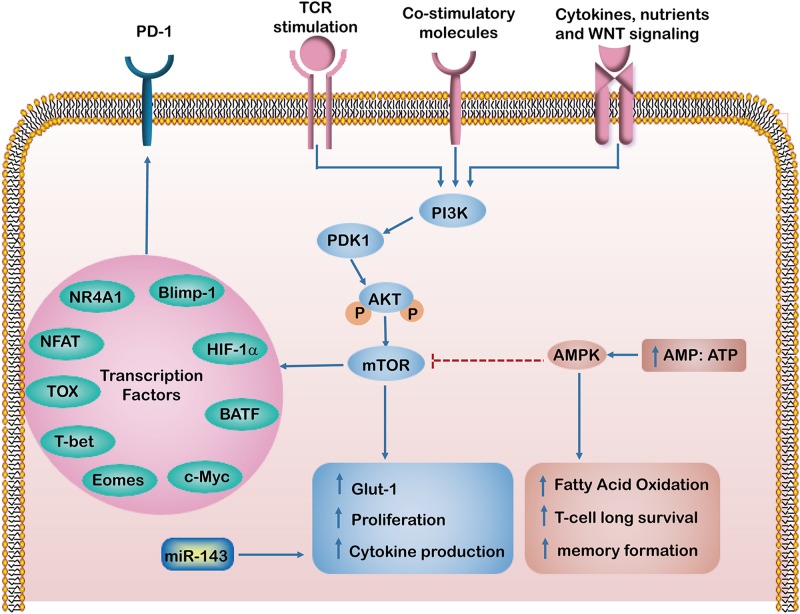
The intrinsic factors regulating T cell dysfunction. In response to T cell receptors (TCRs), co-stimulatory and growth factor cytokines activate PI3K/Akt/mTOR signaling pathways, which induce glucose transporter-1 (Glut-1) expression and enhance T cell proliferation and cytokine production. Activation of mTOR leads to the expression of downstream transcriptional regulators such as HIF-1α and c-Myc. However, an increased AMP to ATP ratio activates AMP-activated protein kinase (AMPK), which in turn inhibits mTOR activity and enhances fatty acid oxidation, which maintains long term T-cell survival and formation of memory T cells. The Transcription factors such as HIF-1α, NR4A1, TOX, Eomes, T-bet, Blimp-1, NFAT and BATF regulate PD-1 expression and have been implicated in T cell exhaustion and dysfunction.

Within the heterogeneous TME, T cells are a major part of the immune infiltrate. The intratumoral T cell population comprises naive, memory, effector and regulatory T cells (Treg) (Hashimoto et al., 2018). Upon stimulation by an antigen, TCRs activate a cell-intrinsic program that guides T cell differentiation into cytotoxic effector cells capable of clearing the antigen. Following the peak of effector cell expansion and the clearance of the specific antigen, most effector T cells die, with the exception of a small number of memory T cells that survive and provide long-term protection against the antigen (Chang et al., 2014). However, when antigen-experienced T cells are chronically exposed to the same antigen, substantial alterations in T cell activation and differentiation may occur, leading to T cell “dysfunction” or “exhaustion” (Wherry, 2011; Schietinger and Greenberg, 2014). A previous study has shown that the tumor-specific T cell dysfunctional exhaustion state is initiated early after tumor initiation and antigen encounter in a murine model (Schietinger et al., 2016). Dysfunctional CD8^+^ T cells are characterized by a loss of effector functions, such as cytotoxicity and proliferation. In addition, the upregulation of immune checkpoints and changes in transcriptional and metabolic molecules have been described as hallmarks of T cell dysfunction (Table 1). For example, the single cell RNA sequencing of tumoral T cells from melanoma and lung cancer indicated that T cells expressed genes such as *PDCD1* and *LAG3* that are associated with T cell dysfunction (Guo et al., 2018; Li H. et al., 2019). Nevertheless, T cell function can be successfully reinvigorated by blocking PD-1 or PD-L1, highlighting the critical role of PD-1/PD-L1 axis in T cell dysfunction. However, activated and functional CD8^+^ T cells can also overexpress PD-1 in cancer patients (Fourcade et al., 2010), and not all PD-1^+^ cells might respond equally to anti-PD-1 therapy (Thommen et al., 2018). It has reported that PD-1^+^CD38^+^CD8^+^ T cells are a population of dysfunctional cells that fail to respond to anti-PD-1 therapy (Verma et al., 2019). Meanwhile, the TME contains a variety of cell types and cytokines (Table 1) that take part in tumor progression, which could contribute to T cell dysfunction (Xia et al., 2019). Therefore, there is growing interest in the identification of the molecular signatures and characteristics that are associated with dysfunctional T cells in cancer (Figure 1).

**TABLE 1:** Core molecular regulation of T cell dysfunction or exhaustion.

**Intrinsic factors**	**Function**	**References**
NR4A	Transcriptional factor that highly expresses in dysfunctional T cells, which can impair anti-tumor effects of T cells and induce PD-1 and TIM-3 expression.	Liu X. et al., 2019
TOX	High-mobility group (HMG)-box transcription factor that regulates the progression of T cell dysfunction and the maintenance of exhausted T cells.	Alfei et al., 2019; Wang et al., 2019d
TCF-1	Transcriptional factor that supports stem-cell function of PD-1^+^ TILs and the formation of exhausted T cell progenitors, which are described that express TCF1 and intermediate amounts of PD-1 (PD-1^int^).	Chen Z. et al., 2019
Eomes	Transcriptional factor that correlates with T cell exhaustion by inducing co-inhibitory molecule B7 superfamily member 1(B7S1) pathway.	Li et al., 2018c
NFAT	A key regulator of T cell activation, can induce exhaustion, which is also the upstream of NR4A and TOX.	Martinez et al., 2015
BATF	Transcription factor that impairs T cell proliferation and cytokine secretion during HIV infection in a pathway downstream of PD-1.	Quigley et al., 2010
Blimp-1	Transcription factor that drives T cells toward a dysfunctional phenotype during chronic LCMV infection.	Hwang et al., 2016
DNMT3A	Epigenetic factor that involves a *de novo* exhaustion-specific DNA methylation pattern, which is important to format the exhausted program.	Ghoneim et al., 2017
mTOR	Metabolic checkpoint that regulates glycolysis via transcription factors including HIF-1α and c-Myc, enhancing the expression of inhibitory receptors in T cells.	Le Bourgeois et al., 2018
TGF-β	Cytokine that induces the expression of TIM-3, PD-1 and CTLA-4 in T cells, and inhibits the secretion of IFN-γ and Granzyme-B.	Wang et al., 2019d
IL-10	Cytokine that suppresses IFN-γ secretion in CD8^+^ TILs. IL-10 blockade enhances the effects of anti-PD-1 therapy in expanding antigen-specific CD8^+^ T cells.	Brooks et al., 2008; Li L. et al., 2019

## Intrinsic Factors That Induced T Cell Dysfunction

### Transcription Factors

It has become increasingly clear that several transcriptional factors, including NR4A1, TOX, Eomes, T-bet, Prdm1 (Blimp-1), NFAT and BATF, regulate the PD-1 expression and are implicated in T cell exhaustion and dysfunction (Wang et al., 2017; Liu X. et al., 2019). For example, NR4A1 was found highly expressed in tolerant or dysfunctional T cells in a mouse model. Overexpression of NR4A1 inhibits effector T cell differentiation, whereas deletion of NR4A1 overcomes T cell tolerance and increases T cell proliferation, enhancing anti-tumor effects. Moreover, expression levels of PD-1 and TIM-3 in T cells were found significantly decreased in NR4A1^–/–^ mice. A mechanistic analysis suggested that NR4A1 is preferentially recruited to binding sites of the transcription factor activator protein 1 (AP-1), where it inhibits effector gene expression by reducing AP-1 function. These findings indicate that NR4A1 is important for inducing T cell dysfunction and represents a promising target for augmenting cancer immunotherapy (Liu X. et al., 2019).

Recently, the high-mobility group (HMG)-box transcription factor TOX was reported as a critical regulator in the progression of T cell dysfunction and the maintenance of exhausted T cells during chronic infection (Alfei et al., 2019). Several studies also showed that TOX may have a role in mediating transcriptional and epigenetic reprograming that are critical for the exhausted CD8^+^ T cells responses in cancer (Khan et al., 2019). Although the formation of effector and memory T cells is not dependent on TOX, the formation of exhausted T cells was failure without TOX. Robust expression of TOX can translate continuous stimulation that induces T cell exhaustion (Khan et al., 2019). Moreover, TOX and TOX2 as well as NR4A family members are highly induced PD-1 and TIM-3 expression in CAR^+^ TILs. TOX and TOX2 deficient CAR^+^ TILs can prevent tumor growth and prolong survival of tumor-bearing mice (Seo et al., 2019). In a mouse model of hepatocellular carcinoma (HCC), TOX was upregulated in exhausted CD8^+^ T cells, impairing their anti-tumor function. The underlying mechanism involves a TOX-induced decrease in PD-1 degradation and promotion of PD-1 endocytic recycling to the cell surface. Knocking down TOX in tumor-specific CD8^+^ T cells promoted the anti-tumor effects of these T cells, exhibiting the synergetic role of anti-PD-1therapy (Wang et al., 2019d).

TCF-1 has been implicated in the formation of memory precursor T cells mediated by Wnt signaling pathway (Jeannet et al., 2010). Similarly, TCF-1 is also required for the stem-like functions of TCF1^+^PD-1^+^ TILs, which were detected in the blood of patients with melanoma and who had responded to checkpoint blockade (Siddiqui et al., 2019). Most notably, TCF-1 enhances Bcl2 expression via c-Mycb and supports the establishment of exhausted T cell progenitors (Chen Z. et al., 2019). These progenitor cells were described that express TCF1 and intermediate amounts of PD-1 (PD-1^int^). These TCF1^+^PD-1^int^ cells give rise to dysfunctional TCF1^–^PD-1^*hi*^ TIM-3^+^ cells, which show resistance to PD-1 blocking therapy. Moreover, TCF1^+^PD-1^int^ cell survival can be boost by upregulating TOX expression (Mann and Kaech, 2019). Scott found that TOX-deficient tumor-specific T cells failed to persist in cancers, and hypothesized that TOX-induced exhaustion serves as a negative feedback mechanism that prevents activation-induced T cell death and overstimulation of antigen-specific T cells (Scott et al., 2019). These findings suggest that TOX may play a two-blade function in T cell dysfunction or exhaustion.

T-bet and Eomes were found to operate in contrasting ways to facilitate the effector versus memory CD8^+^ T cell fates (Chang et al., 2014). Enhanced T-bet expression fosters effector differentiation of antigen-specific CD8^+^ T cells toward the terminally differentiated fate. In contrast, Eomes is highly expressed in memory T cells and is considered important for the maintenance of memory T cells (Knudson et al., 2017). Notably, recently studies identified high expression levels of Eomes in exhausted CD8^+^ T cells during chronic lymphocytic choriomeningitis virus (LCMV) infection. Interestingly, the CD8^+^ T cells producing high levels of Eomes also expressed high levels of PD-1. These Eomes^hi^PD-1^hi^ CD8^+^ T cells co-expressed other inhibitory receptors and displayed limited proliferative capacity (Li et al., 2018b). In addition, Eomes is directly involved in exhaustion of CD8^+^ TILs via the co-inhibitory molecule B7 superfamily member 1(B7S1) pathway (Li et al., 2018c). However, how increased expression of Eomes promotes CD8^+^ T cell exhaustion remains elusive.

Studies have shown that NFAT, a key regulator of T cell activation, can induce hyporesponsiveness (anergy and exhaustion) in both CD4^+^ and CD8^+^ T cells, if it does not bind AP-1 transcription factors (Martinez et al., 2015). Moreover, TOX and NR4A are important for the transcriptional program of CD8^+^ T cell exhaustion downstream of NFAT (Seo et al., 2019). Intriguingly, BATF, a transcription factor of the AP-1 family, was found to impair T cell proliferation and cytokine secretion during HIV infection in a pathway downstream of PD-1 (Quigley et al., 2010). Similarly, Blimp-1 was found to drive T cells toward a dysfunctional phenotype during chronic LCMV infection (Hwang et al., 2016). Further identifying how these transcription factors are integrated together to mediate CD4^+^ and CD8^+^ T cell exhaustion or dysfunction will provide molecular insights into T cell responses and immunity.

### Epigenetic Factors

Emerging evidence indicates that epigenetic states and chromatin landscapes are closely associated with the functional state of dysfunctional or exhausted CD8^+^ T cells, which are abnormally expressed PD-1 (Pauken et al., 2016; Sen et al., 2016; Kartikasari et al., 2018). Epigenetic components including DNA methylation and histone modifications could control PD-1 expression and T cell exhaustion. For example, DNA methylation enzymes such as DNMT1 and DNMT3B are significantly upregulated in exhausted T cells (Schietinger et al., 2016). Meanwhile, DNA methyltransferase 3A (DNMT3A) has been demonstrated to functionally establish a *de novo* exhaustion-specific DNA methylation pattern. Inhibition of DNMT3A in these T cells can promote their differentiation toward memory cells. Critically, observations from studies of chronic viral infections indicated a critical role for the demethylation at the promoter region of PD-1 locus in mediating T cell dysfunction (Ghoneim et al., 2017). Inhibition of DNA methylation leads to a revitalized effect on the function of exhausted T cells.

The most widely studied histone lysine methylation sites in T cells are histone 3 lysine 4 (H3K4), and H3K27. H3K4 methylation is associated with transcriptional activation, and H3K27 trimethylation is associated with the repression of genes important for T-cell differentiation and survival (Liu H. et al., 2019). When TCR stimulation and IL-6 or IL-12 treatment were combined, both H3K4^me1^ and H3K27 acetylation were contributed to increased PD-1 expression (Bally et al., 2016). Moreover, overexpression of miR-155 significantly enhances polycomb repressor complex 2 (PRC2), which restrains T cell exhaustion and sustains CD8^+^ T cell antitumor responses (Ji et al., 2019). Additionally, EZH2 is a catalytic subunit of PRC2 that can alter gene expression by trimethylating H3K27 (Zhao et al., 2016; He et al., 2017). In a recent study, EZH2 was found to control the polyfunctionality and differentiation of effector T cells (He et al., 2017). Interestingly, inhibition of EZH2 in ovarian and colorectal cancer patients resulted in increased of CXCL9 and CXCL10 production and augmented the infiltration of T cells that eliminate tumors (Nagarsheth et al., 2016; Jones et al., 2018). EZH2 represses the expression of tumor suppressor genes in various cancer cells, thereby promoting cell invasion and driving tumor progression (Bohrer et al., 2010; Hayashi et al., 2011). Thus, EZH2 may be a promising target for cancer immunotherapy (Wang et al., 2019c). Meanwhile, treatment with JQ1-α specific inhibitor of the histone acetylation reader bromodomain-containing protein 4 (BRD4)-results in decreased PD-L1 expression on tumor cells and macrophages, which is correlated with an increase in antitumor T cell activity (Zhu et al., 2016). These findings indicate that pharmacological manipulation of epigenetic mechanisms can alter T cell exhaustion or dysfunction in a clinically relevant manner. Thus, therapies employing hypomethylating agents and PD-1 blockade are a promising strategy in cancer patients.

### Metabolic Factors

The metabolic program is a set of biochemical reactions that allows T cells to acquire and utilize nutrients necessary for their survival, proliferation, and functions (Chang et al., 2015). It has been shown that effector T cells use glycolysis for anabolic metabolism for their growth and proliferation. In contrast, memory T cells switch to a non-proliferative form of metabolism, using FAO as a predominant metabolic program and obtaining ATP mainly via OXPHOS (Pearce et al., 2013). Interestingly, the link between antigenic stimulation and metabolic pathway activation appears to be altered in dysfunctional T cells (Le Bourgeois et al., 2018; Sugiura and Rathmell, 2018). Recently, metabolic checkpoints (e.g., AMPK, Myc, HIF-1α and mTOR) that control T cell differentiation have been highlighted as a novel therapeutic targets for immune modulation (Chang and Pearce, 2016). AMPK is a heterotrimeric serine/threonine kinase complex that senses the intracellular AMP/ATP ratio. Activated AMPK can enhance FAO and simultaneously inhibit glucose and mTOR activity. This results in maintaining long-term T cell survival and memory formation (Ma et al., 2017). A previous study demonstrated that the abrogation of phosphoenolpyruvate carboxykinase (Pck1)–glycogen–pentose phosphate pathway (PPP) decreases GSH/GSSG ratios and increases levels of ROS levels, leading to impairment of memory CD8^+^ T cell formation (Ma et al., 2018). Our previous work demonstrated that miR-143 enhances the anti-tumor effects of CD8^+^ T cells by promoting memory T cell differentiation and metabolism reprograming by inhibiting of glycolysis targeting glucose transporter-1 (Glut-1) (Zhang et al., 2018). TCR signaling, together with costimulatory molecules and growth factor cytokines, activates phosphatidylinositide 3 kinase (PI3K)/Akt/mTOR signaling pathways, which induce Glut-1 and enhance T cell proliferation and cytokine production (Salmond, 2018). Furthermore, activation of the mTOR pathway and engagement of glycolysis lead to the expression of downstream transcriptional regulators such as HIF-1α and c-Myc, enhancing the expression of inhibitory receptors on T cells (Le Bourgeois et al., 2018). Meanwhile, suppression of Akt and mTOR is required for augmenting activity of the transcription factor FoxO1. Importantly, FoxO1 sustains PD-1 expression, which promotes the differentiation of terminally exhausted T cells (Staron et al., 2014). Therefore, these intrinsic metabolic factors regulate T cell metabolism and activate pathways involved in effector function and exhaustion.

## Extrinsic Factors: Tumor Microenvironment

**FIGURE 2:**
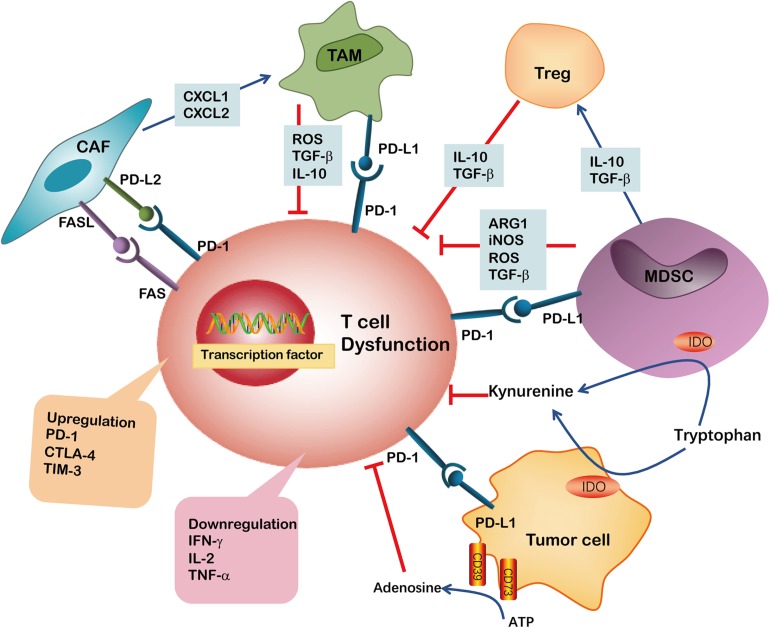
Immunosuppressive cells or factors have been implicated in CD8^+^ T cell dysfunction in TME. The ARG1, iNOS, TGF-β and ROS are secreted by MDSCs or TAMs and induce CD8^+^ T cell dysfunction. Both MDSCs and tumor cells may suppress CD8^+^ T cell proliferation through IDO hydrolyzation of tryptophan in the presence of IFN-γ. Kynurenine inhibits CD8^+^ T cell activation. MDSCs may additionally produce immunosuppressive cytokines like IL-10, TGF-β and induce Tregs. The upregulation of PD-L1 on MDSCs, TAMs and tumor cells induced CD8^+^ T cell exhaustion by binding to PD-1 on T cells. Tumor cells also express CD39 and CD73 on their surface, facilitating the metabolism of extracellular ATP into AMP and finally into adenosine, which induce CD8^+^ T cell dysfunction. CAFs are involved in impairing anti-tumor T cell responses by secreting chemokines such as CXCL1 and CXCL2 to tumors and polarizing them toward the M2 phenotype. Furthermore, the expression of PD-L2 or FASL on CAFs bind to corresponding PD-1 and FAS receptors, respectively, causing CD8^+^ T cell dysfunction.

Apart from T cell self-regulation, the interaction between other cells or cytokines in the TME is another important factor that induces T cell dysfunction. Various types of cancers and cytokines compose the TME, including tumor cells, immunosuppressive cells, stromal cells, IL-6 and IL-10, to name a few, which collectively form a complex of immunosuppressive network. These TME components exert potent effects on limiting T cell differentiation and driving T cell dysfunction. In addition, tumor cells and immunosuppressive cells within TME produced highly reactive soluble oxygen and toxic metabolites, which inhibit T cell responses (Maimela et al., 2019). Thus, it is challenging to precisely define the relative contribution of these potential extrinsic factors to T cell function and differentiation in the TME (Figure 2).

## Myeloid Derived Suppressor Cells

Myeloid-derived suppressor cells (MDSCs) play pivotal roles in promoting tumor progression and contribute to immunosuppressive function (Ostrand-Rosenberg, 2010; Li et al., 2018e). MDSCs are a heterogeneous group of pathologically activated immature myeloid cells in the TME. The various mechanisms implicated in MDSC-mediated immune suppression include the release of high levels of arginase (ARG)-1, inducible nitric oxide synthase (iNOS), reactive oxygen species (ROS) and cyclooxygenase-2 (COX2) (Chen J. et al., 2019). For example, ARG-1 and iNOS, either separately or in combination, are used by MDSCs to impede CD8^+^ T cells response to antigens. Indeed, expression of ARG-1 has been reported to decrease CD3ζ-chain biosynthesis, thus impairing T cell function (Rodriguez et al., 2002). In addition, NO alone can suppress CD8^+^ T cells by inhibiting the phosphorylation and activation of JAK3 and STAT5 transcription factors, as well as inducing T-cell apoptosis (Wu et al., 2015). Elevated levels of ROS in the TME was involved in limiting T cell growth, differentiation, and ultimately promoting the exhaustion of T cells. ROS produced by MDSCs and other cells may interact with T cells and cause oxidative stress which may induce CD8^+^ T cell hypo-responsiveness in cancer (Chen et al., 2016).

Interestingly, MDSCs produce high levels of IDO, which catabolizes tryptophan and generates kynurenine. Depletion of tryptophan and induction of kynurenine lead to blockade of clonal expansion of activated T cells. Experimental studies indicate that IDO hydrolyzation of tryptophan represents an important mechanism by which MDSCs suppress proliferation and survival of tumor infiltrating CD8^+^ T cells and CD8^+^ T cells homed to the lymph nodes. Other reports demonstrated that STAT3-dependent IDO expression mediates immunosuppressive effects of MDSCs in breast cancer, in which MDSCs dramatically inhibit the proliferation of CD8^+^ T cells and their production of IFN-γ (Yu et al., 2013). Data from our previous studies indicate that CD11b^+^CD33^+^ MDSCs in tumor tissues from NSCLC patients express surface ectonucleotidases CD39 and CD73. Moreover, TGF-β stimulates CD39 and CD73 expression, thereby inhibiting autologous CD8^+^ T cell proliferation and function (Li et al., 2017). Thus, MDSCs play an important role in repressing CD8^+^ T cell proliferation thus inducing CD8^+^ T-cell exhaustion in TME.

## Tumor-Associated Macrophages

Macrophages play a critical role in innate immunity and are responsible for defending the host against foreign pathogens. They can be further classified into pro-inflammatory M1 or anti-inflammatory M2 macrophages. M1 cells are characterized by the high expression of various pro-inflammatory cytokines and contribute to promoting the Th1 response. They also have strong microbicidal and tumoricidal activity (Sica and Mantovani, 2012; Ambade et al., 2016). M2 cells, also known as alternatively activated macrophages, are activated by Th2 cytokines (e.g., IL-4, IL-10, and IL-13) and secret high levels of anti-inflammatory cytokines such as IL-10, and TGF-β. M2 macrophages primarily contribute to immune-suppression and favor tumor promotion (Liu S. et al., 2019).

Tumor-associated macrophages (TAMs) are generally characterized as an M2-like macrophage phenotype in TME. They can be identified by the expression of CD163, CD204 or CD206 in human derived TAMs, and F4/80, CD163, CD206, ARG1 or Ym1 in murine-derived TAMs (Cassetta et al., 2016; Chen Y. et al., 2017; Benner et al., 2019). TAMs often accelerate the progression of untreated cancer and negatively influence the efficacy of anticancer drugs, including checkpoint blockade immunotherapies. Therefore, TAMs are shown to be closely correlated with a poor prognosis of patients with cancer. Li and colleagues demonstrated that TAMs-secreted IL-10 promotes cancer stem cell-like properties and tumor growth in NSCLC; High levels of IL-10 are associated with a poor prognosis of NSCLC patients (Yang and Zhang, 2017). TAMs can induce immunosuppression mainly through several ways: (1) TAMs may induce the expression of PD-L1 in monocytes, which binds to PD-1 on the surface of CD8^+^ T cells, inducing T cell exhaustion; (2) TAMs secret numerous immunosuppressive cytokines and factors, including IL-10, TGF-β and ROS, which induce CD8^+^ TIL exhaustion and dysfunction; and (3) TAMs can directly inhibit CD8^+^ T cells cytotoxicity through the depletion of the amino acids, such as L-arginine and tryptophan. In addition, these functions indicate that TAMs produce high levels of IDO to inhibit CD8^+^ T cells cytotoxicity. Collectively, TAMs are a highly active subset of immunosuppressive cells promoting tumor survival and immune evasion (Jiang et al., 2015; Yang and Zhang, 2017).

Efforts are underway to either deplete M2 cells or convert the M2 phenotype into M1 cells (inflammatory) in most tumors. In a mouse model of ovarian cancer, it was shown that tumor rejection by CAR-T cells required the presence of M1 macrophages, suggesting that tumor-reactive T cells were not sufficient to completely eliminate the tumor on their own (Yeku et al., 2017). We have recently reported that M1 macrophages converted from M2 macrophages by *Pseudomonas aeruginosa*-mannose-sensitive hemagglutinin (PA-MSHA) can enhance the anti-tumor immune response. This effect primarily relies on activation of toll like receptor 4 (TLR4) (Yang et al., 2015).

## Cancer Associated Fibroblasts

Cancer-associated fibroblasts (CAFs), the most abundant stromal population, secrete immunomodulatory factors in TME and are emerging as suppressive mediators of T cell immunity. CAFs can recruit myeloid cells to tumors via the secretion of chemokines such as CXCL1 and CXCL2 to tumors. They can also polarize these recruited myeloid cells toward the M2 phenotype (Zhen et al., 2017). Similarly, CAFs can further bolster the immunosuppressive TME by recruiting MDSCs and Tregs. Thus, the crosstalk between CAFs and other cells contributes to the immunosuppressive TME. Other studies revealed a new biological function for CAFs, showing that these cells directly suppress anti-tumor T-cell responses by a mechanism dependent on immune checkpoint activation. One of the underlying mechanisms was the upregulation of FAS/FASL and PD-1/PD-L2 on CD8^+^ T cells and CAFs, respectively, which drives the dysfunction of tumor-specific CD8^+^ T cells (Lakins et al., 2018). Targeting CAFs or CAFs-related pathways can be considered as a powerful strategy for attenuating stromal barriers and promoting cancer immunotherapy.

## Soluble Mediators

Immunosuppressive cytokines in TME can be produced by tumor cells, MDSCs or CAFs and are crucial factors that mediate T-cell exhaustion and dysfunction (Chen J. et al., 2017). For example, the TGF-β signaling pathway plays an important role in tumor suppression and paradoxically, plays a role in tumor promotion. In general, TGF-β mediates tumor suppression via the inhibition of cancer cell proliferation and induction of cancer stem cell senescence by diminishing their self-renewing capability during the early stages of tumor development. Additionally, TGF-β promotes tumor progression and metastasis through the modulation of immune responses in later stages (Colak and Ten Dijke, 2017). TGF-β derived TAMs exert its function by inducing TIM-3, PD-1 and CTLA-4 expression in T cells and inhibiting IFN-γ and Granzyme-B secretion in a dose-dependent manner. Treatment with anti-TGF-β antibody restored the impaired T cell cytotoxic function in MPE. Furthermore, inhibiting TGF-β signaling in CD8^+^ T cells using dominant negative receptors can improve the function of exhausted cells (Wang et al., 2019b).

IL-10 in TME is primarily secreted by cancer cells, TAMs, natural killer cells (NK) and CD4^+^ Tregs (Landskron et al., 2014). A study of patients with ovarian carcinoma demonstrated that the tumor-infiltrating follicular regulatory T (Tfr) cells exhibit significantly upregulated IL-10 expression, which is negatively associated with IFN-γ secretion in CD8^+^ TILs (Li L. et al., 2019). In chronic viral infections, PD-1 blockade augments IL-10R expression by antigen-specific CD8^+^ T cells, thereby increasing their sensitivity to the immunosuppressive effects of endogenous IL-10. Conversely, IL-10 blockade strengthens the effects of PD-1 blockade in expanding antigen-specific CD8^+^ T cells and reinforcing their function. Thus, IL-10 and PD-1 pathways act synergistically through distinct pathways to suppress T cell survival and function (Brooks et al., 2008).

## Cancer Immunotherapy and T Cell Dysfunction in the TME

As the pivotal player in the adaptive immune system, T cells can recognize and eliminate tumor cells. However, tumor cells evade from the immune attack once the T cells enter dysfunctional state. The emergence of engineered T cells as a form of cancer therapy marks the beginning of a new era in medicine, providing a transformative way to combat tumors (Mikkilineni and Kochenderfer, 2017). To date, CD19-targeted CAR T-cell therapy has been largely successful in hematological malignancies, showing up to 90% complete response in relapsed or treatment-refractory acute lymphoblastic leukemia (ALL) patients (Maude et al., 2014). However, despite extensive research, the efficacy of CAR-T cell therapy on controlling solid tumors is limited effects due to the influence of TME (Yu et al., 2017; Li et al., 2018d; Yan et al., 2019). Studies suggest that solid tumors may induce hyporesponsiveness of CAR-T cells (Irving et al., 2017; Martinez and Moon, 2019). Three CAR-T cell trials that targeted IL13Rα2, Her2/CMV and EGFRvIII showed poor T cell persistence and an inability to prolong overall survival of patients with glioma (Brown et al., 2016; Ahmed et al., 2017; O’Rourke et al., 2017; Migliorini et al., 2018). Compared with pre-infusion tumor tissues, post-CAR-T cell infusion tumor specimens show markedly upregulated expression of many immunosuppressive molecules, particularly IDO1 and Foxp3, and in some cases, IL-10, PD-L1, and/or TGF-β (O’Rourke et al., 2017). Lim and June (2017) suggested that the TME of solid tumors is hostile for CAR-T cells, reporting that even though CAR-T cells successfully penetrate into the tumor, they are exposed to numerous suppressive factors and tumor-associated stromal cells, which contribute to limiting their function. Additionally, these infused CAR-T cells express high levels of PD-1, T-cell immunoglobulin domain and mucin domain protein 3 (TIM3) and CTLA-4, indicating that they become exhausted or dysfunctional cells (Lim and June, 2017).

Recent studies have identified the crucial role that NR4As play in mediating exhaustion of CAR-T cells. In the NR4A-knocked out mice, CAR-T cells demonstrated low expression levels of inhibitory receptors and increased anti-tumor activity *in vivo* (Chen J. et al., 2019). Other studies have demonstrated the critical roles of that metabolic barriers play in the TME. Low glucose levels in the TME produce particular challenges for memory CAR-T cells. Thus, novel strategies are needed to assist CAR-T cells in overcoming the immunosuppressive microenvironment presented of many solid tumors.

Cancer neoantigens are derived from random somatic mutations in tumor tissues and are attractive targets for cancer immunotherapies (Chu et al., 2018). Neoantigen-based personalized cancer vaccines have recently shown marked therapeutic potential in both preclinical and early phase clinical studies. Although the number of patients with advanced melanoma treated by neoantigen vaccines is small, results from several phase I clinical trials are quite encouraging. Reportedly, neoantigen-pulsed dendritic cells may induce neoantigen-specific T-cell responses in these patients. A phase I/Ib glioblastoma trial also verified that the circulating polyfunctional neoantigen-specific CD4^+^ and CD8^+^ T cell responses were generated in these patients (Ott et al., 2017). However, cancer types with a low mutation burden may not be eligible for this vaccine therapy. Meanwhile, the complicated TME possesses numerous immunosuppressive mechanisms that result in immune escape.

Importantly, tumor cell clones can generate tumors that recapitulated T cell-inflamed and non-T-cell-inflamed TMEs upon implantation in immunocompetent mice, with characteristic patterns of infiltration by immune cell subsets. CXCL1 was identified as a determinant of the non-T-cell-inflamed microenvironment, and ablation of CXCL1 promoted T cell infiltration and sensitivity to immunotherapy (Li et al., 2018a). Furthermore, lower expression levels of co-stimulatory molecules and higher expression levels of co-inhibitory receptors, such as PD-L1, have been shown to be correlated with T-cell dysfunction. Therefore, many efforts are currently focused on addressing challenges in the development of neoantigen-based cancer vaccines for wide clinical applications. Notably, two melanoma patients that experienced disease relapse after successful neoantigen vaccine treatments, and later, achieved complete response after subsequent anti-PD-1 antibody treatment (Ott et al., 2017).

One of the most popular and successful strategies to combat T cell exhaustion is the use of checkpoint inhibitors. The ICB such as anti-PD-1, anti-PD-L1 and anti-CTLA-4 are currently approved by the U.S. FDA for various of caner types. However, the response rate of ICB therapy is less than 30% in solid tumors. First, PD-1 expression levels have a distinct role in contributing to T cell dysfunction or resisting to PD-1 blockade. While intratumoral PD-1^high^ CD8^+^ subsets share the properties of co-expression of inhibitory receptors and loss of effector function, these populations secreted high levels of CXCL13, which can recruit immune cells to TME. Moreover, the presence of PD-1^high^ T cells was strongly predictive for clinical outcome in a small number of NSCLC patients treated with anti-PD-1 (Thommen et al., 2018). Second, cancers that are non-responsive to checkpoint blockade therapies usually already have decreased numbers of T cells infiltrating their tumors. Infiltrating T cells often co-express multiple inhibitory markers, and expression of the corresponding ligands is evident in tumor cells. In addition, factors beyond tumor genomics influence cancer development and therapeutic responses, including host factors such as the gastrointestinal (gut) microbiome and obesity (Gopalakrishnan et al., 2018; Popovic et al., 2018; Wang et al., 2019e). For example, the frequency of CD8^+^ TILs expressing PD-1and TIM3 is higher in the diet-induced obese mice (DIO) than that in control mice. Anti-PD-1 monotherapy had minimal to no effect on control mice but significantly reduced tumor burden and significantly improved the survival of DIO mice. It remains to be clinically described whether the environment in the obese state results in greater T cell function once checkpoint blockade is applied (Wang et al., 2019e). More importantly, understanding the relationship between heterogeneous dysfunctional T cells and the TME may significantly impact on the success of therapies like checkpoint blockade and could lead to the production of more functional CAR-T cells.

## Reversing T Cell Dysfunction by Combination Cancer Immunotherapy

**FIGURE 3:**
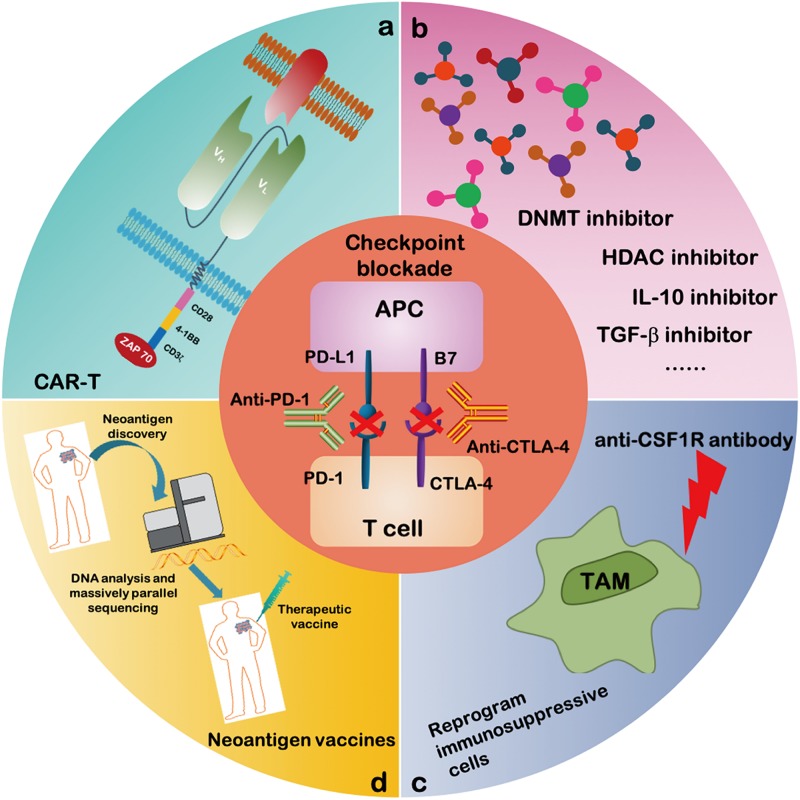
Combinatorial therapeutic strategies to reverse the T cell dysfunction. A growing number of studies propose to evaluate the efficacy of immune checkpoint blockade antibodies together with: **(a)** CAR-T cell therapy; **(b)** inhibitors of soluble mediators targeting IL-10, TGF-β, DNA methyltransferase and histone deacetylase; **(c)** CSF1R antibody targeting activating receptors on TAMs to inhibit the polarization of TAM; and **(d)** neoantigen vaccines.

Great efforts have been made to characterize the intrinsic properties of dysfunctional T cells. Additionally, transcriptional regulators as well as metabolic and epigenetic factors have been investigated as possible targets to improve the anti-tumor efficacy of immunotherapies (Figure 3). New treatment strategies employing epigenetic drugs and immune checkpoint blockade therapies have been investigated in an effort to reverse T cell dysfunction. For instance, in a mouse model of epithelial ovarian cancer, the DNA methyltransferase and histone deacetylase inhibitors (DNMTi and HDACi, respectively) can reduce the immunosuppressive microenvironment through type I IFN signaling and improve response to anti-PD-1 therapy. Addition of HDACi and DNMTi 5-azacytidine (AZA) enhances the modulation of the immune microenvironment, specifically increasing T cell activation and reducing the percentage of macrophage *in vivo* (Stone et al., 2017).

Recent studies have also provided evidence that classical immune checkpoints can interact with metabolic checkpoints. In a mouse sarcoma model, glucose consumption by tumors metabolically restricts T cells, leading to their dampened mTOR activity and facilitating cancer progression. It was also discovered that PD-L1 blockade can act directly on tumor cells to inhibit mTOR activity, increasing extracellular glucose availability (Chang et al., 2015). These findings suggest that immune checkpoint blockades counteract T cell dysfunction not only by preventing intrinsic T-cell inhibitory signals but also by increasing T cell metabolic fitness. Meanwhile, the AMPK activator metformin, a first-line treatment drug for type 2 diabetes, was reported to have anti-cancer activity. In a mouse model, metformin was found to increase the number of CD8^+^ TILs and protected them from apoptosis and exhaustion characterized. Thus, a direct effect of metformin on CD8^+^ T cells is critical for protecting against the T cell exhaustion in TME. Thus, the combined use of metformin and cancer vaccines can improve TILs multifunctionality (Eikawa et al., 2015).

However, whether the combination of ICB and metformin can restore the dysfunctional T cells remains unclear. Transcriptional profiling of dysfunctional T cells revealed a set of transcription factors that are altered in expression compared with effector or memory T cells. Indeed, a growing list of transcription factors that can regulate the expression of inhibitory receptors has been identified, highlighting potential targets for immunotherapy. Two reports have identified NR4A transcription factors as key mediators of T cell function and demonstrated that NR4A deficiency leads to the downregulation of PD-1, which is functionally similar to the effects of PD-1 blockade (Chen J. et al., 2019; Liu X. et al., 2019). Thus, inhibiting the function of NR4A in TILs or CAR-T cells could be a promising strategy in cancer immunotherapy, similar to combination therapies with ICB against CTLA-4 or GITR (glucocorticoid-induced tumor necrosis factor receptor–related protein) antibodies. Additionally, TOX has been defined as an important transcription factor in regulating T cell exhaustion. Possibly, reducing TOX expression in combination with anti-PD-1 therapy can potentially provide a more effective strategy of abrogating the TOX-dependent pathway of CD8^+^ T cell exhaustion. Emerging data from a clinical study reported limited clinical activity for anti-GITR monotherapy but potentially promising data for the combination therapy. Combination treatments aimed at PD-1 inhibition and activation of GITR, decrease CD8^+^ T cell dysfunction and induce a highly proliferative precursor effector memory T cell phenotype (Wang et al., 2018). Monotherapy with CTLA-4 leads to disease control in 20–28% of patients with metastatic melanoma. However, the maintenance of T cell responses triggered by anti-CTLA-4 alone is in most cases insufficient to successfully eradicate tumors, and durable long-term complete remissions (CRs) are seen in a minority of patients. Similarly, adoptive transfer of peripheral blood-derived antigen-specific cytotoxic T cells (CTLs) alone is generally insufficient to eliminate tumors, whereas IL-21-primed CTLs with characteristics of a long-lived memory phenotype may enhance T cell survival after infusion to patients. Thus, the anti-CTLA-4 combined with IL-21-primed CTLs results in long term T cell persistence and durable anti-tumor function (Chapuis et al., 2016a, b).

Importantly, cancer immunotherapies aim to reinvigorate T cell function as well as target immunosuppressive and tumor-promoting pathways mediated by TME (Figure 3). Several specific strategies that target TME are being investigated in combination with ICB therapies in order to improve T cell mediated immunotherapy. Recently, there has been a significant new interest in using macrophage modulators to optimize TAMs. An anti-CSF1R antibody was shown to reprogram TAM polarization and improve the responses to ICB therapy in pancreatic cancer (Cassetta and Kitamura, 2018). Other strategies are focused on inhibiting MDSC function and depleting and/or reprograming MDSCs to enhance the efficacy of checkpoint agents. A clinical trial showed MDSC frequencies as potential biomarkers and reported on their correlation with clinical outcomes of melanoma patients treated with ipilimumab (Meyer et al., 2014). Interestingly, a host of cytokines released by immune and tumor cells have been found to negatively contribute to immunosuppression and have therefore been targeted toward reprograming the immunosuppressive TME. Thus, a combined treatment of IL-10 or TGF-β inhibitor(s) with ICB represents a promising strategy for immunotherapy strategy (Zarour, 2016).

## Conclusion

CAR-T cells, neoantigen vaccines and immune checkpoint-modulating agents have increasingly been proven successful in driving antitumor immune responses. Despite these rapid advances in cancer immunotherapy, enormous challenges remain for the future development of cancer therapy for wide clinical applications. Most clinical and preclinical studies using immunotherapy have been focused on T cell exhaustion and dysfunction in TME. In this review, we discussed the unique transcriptional programs and the metabolic and epigenetic factors underlying tumor-induced T cell dysfunction, with the hope that a clearer understanding of TME may enable the development of novel targeted therapeutics, improving the efficacy of immunotherapies. Moreover, the following aspects should be given more attention, (1) identification of mechanisms that convert immunologically cold tumors to T cell rich hot tumors; (2) agents or strategies that reverse T cell exhaustion, and/or reprogram an otherwise immunosuppressive TME must be employed together with immune checkpoint modulators to achieve a robust and durable clinical response; and (3) utilizing RNA sequencing or NanoString tumor expression profiles, to identify gene signatures of T cell dysfunction and predict the outcome of patients treated with checkpoint modulators. This will aid in identifying new targets and advance our fundamental understanding of new targets or the optimal combination therapies for cancer patients.

## Author Contributions

ZZ, SL, and YZ (sixth author) conceptualized this review, decided on the content, and wrote the manuscript. ZZ and SL prepared the figures. BZ, LQ, and YZ (fifth author) revised this review. All authors approved the final version of the manuscript and agreed to be accountable for all aspects of the work.

## Conflict of Interest

The authors declare that the research was conducted in the absence of any commercial or financial relationships that could be construed as a potential conflict of interest.

## Abbreviations

ARG-1: arginase-1
CAFs: cancer-associated fibroblasts
CAR: chimeric antigen receptor
CAR-T: chimeric antigen receptor-engineered T cells
ETC: endogenous peripheral blood-derived T cells
EZH2: enhancer of zeste homolog 2
FAO: fatty acid oxidation
Glut-1: glycosis transporter-1
ICB: immune checkpoint blockers
IDO: indoleamine 2,3-dioxygenase-1
IFN- γ: interferon- γ
IL-2: interleukin-2
iNOS: inducible nitric oxide synthase
LAG-3: lymphocyte activation gene 3
MDSCs: myeloid derived suppressor cells
MPE: malignant pleural effusion
NSCLC: non-small cell lung cancer
OXPHOS: oxidative phosphorylation
PA-MSHA: *Pseudomonas aeruginosa*-mannose- sensitive hemagglutinin
PD-1: programed cell death 1
PD-L1: programed cell death ligand 1
ROS: reactive oxygen species
TAMs: tumor-associated macrophages
TCR: T cell receptor
TCR-T: TCR-engineered T cells
TGF- β: transforming growth factor- β
TIGIT: immunoreceptor tyrosine-based inhibitory motif domain
TIL: tumor infiltrated lymphocytes
TIM-3: T-cell immunoglobulin domain and mucin domain protein 3
TLR4: toll like receptor 4
TME: tumor microenvironment
TNF- α: tumor necrosis factor- α
Tregs: T regulatory cells.
